# The unraveling of Amsterdam’s unitary rental system

**DOI:** 10.1007/s10901-018-9601-x

**Published:** 2018-03-06

**Authors:** Robbin Jan Van Duijne, Richard Ronald

**Affiliations:** 1 Centre for Urban Studies, University of Amsterdam, Amsterdam, The Netherlands; 2 School of Social Policy, University of Birmingham, Birmingham, UK

**Keywords:** Private renting, Social renting, Unitary rental market, Social and economic polarization, Dualisation

## Abstract

The Netherlands has traditionally been considered an iconic ‘unitary’ rental housing market in which social and private sectors directly compete. More recently however, this unitary market has been undermined by changes in the status of housing associations, the privatization of social housing stock and the promotion of home ownership. It has subsequently been suggested that the Netherlands is drifting toward a ‘dualist’ system in which social and private sectors are critically unequal. This paper takes on this claim, providing, on the one hand, palpable evidence of the waning influence of the unitary housing system in the Netherlands and, on the other, a deeper examination of processes of dualisation as well as the outcomes. We focus on Amsterdam, where housing privatization has been most intense. We specifically draw on a geospatial analysis of changing tenure distributions at the neighbourhood level as well as a household analysis of the shifting profile of tenants and home owners to show how the unitary rental market, which helped establish Amsterdam as an iconic ‘just city’, has been unraveling. We demonstrate the relevance of the unitary/dualist model to understanding contemporary urban processes, especially those featuring social and economic polarization.

## Introduction

In recent decades, the Netherlands has undergone dramatic housing sector restructuring with home ownership rates increasing from 45 to 60% since 1990, primarily at the expense of the social rental sector (CBS 2014). It has thus been suggested that the Netherlands is drifting towards a ‘dualist based housing system’: one where the private market—mainly owner occupation—is considered the primary means to house the population, with social rental housing ring fenced for categories of poor and marginalized (see Elsinga et al. 2008). While the suggestion of a drift towards *dualisation* was initially posited in the pre-crisis era (Van Kempen and Priemus 2002; Elsinga et al. 2008), since the late-2000s, and especially the Global Financial Crisis (GFC), housing marketization has intensified and social housing become a more direct target of deregulatory intervention.

This article addresses recent housing sector restructuring, focusing at the urban level. We consider the example of Amsterdam, where the share of home ownership increased from 11 to 30% between 1995 and 2015, while the ratio of social rental housing declined from 58 to 44% (O&S 2015). We take on Amsterdam, not only because dualisation appears to have manifest more intensely, but also because of the long established influence of its large social rental sector in sustaining social equality in the city (Fainstein 2000, 2010). A particular concern of our empirical analysis is the extent to which private and social housing sectors still compete on equal terms (as in unitary markets), as well as how dualisation is contributing to urban and social change.

The concepts of unitary and dualist systems have been influential in comparative housing studies for more than two decades (e.g., Kemeny 1995; Kemeny et al. 2005; Elsinga et al. 2008; Hoekstra 2009; Czischke 2009; Priemus 2014; Stephens et al. 2008). Nonetheless, contributions have customarily focused on national level policy developments and rarely taken urban spatial differentiation into account. They have, furthermore, reflected poorly on the outcomes in terms of socioeconomic polarization. This paper illustrates how changes in housing and rental systems have distinct socio-spatial effects at the urban level. As dualist systems characteristically deepen inequalities, we can begin to associate this process more closely with intensified segregation, marginalization and socio-economic polarization. The paper also opens up underexplored theoretical avenues of rental system analysis in housing studies. While contributions in this field generally put greater emphasis on path dependency of one rental system, we focus specifically on the transition from one rental system to another, and argue for the theoretical usefulness of this approach.

Our analysis proceeds in a number of parts. We begin by consider the significance of housing systems, zooming in on the linkages between housing, society, and urban space. We also elaborate on how and why unitary rental systems in countries like the Netherlands have recently come ‘under threat’ (e.g., Boelhouwer and Priemus 2014; Elsinga et al. 2008; Stephens et al. 2008). In the following sections we move from a national to a city level analysis. Drawing on the findings from Amsterdam, we deal with the particular advance of home ownership as well as emerging differences between social and private renting and renters. We specifically explore dualisation in Amsterdam’s social and private housing markets in spatial terms as well as in relation to rent levels and tenant profiles. In the final section we reflect on the salience of our Amsterdam findings to core debates in urban geography, as well as the implications for the development of Kemeny’s unitary/dualist approach in analyses of urban systems.

## Housing systems, tenure and society

A housing system represents far more than just the way each society regulates the production and consumption of shelter. For Kemeny (1995), they are the outcome of, and also contribute to, an overall political constellation in each context. Kemeny further argues that different groups of societies pursue different tenure strategies, specifying the major principles that underlay divergent approaches. At the heart of this analysis is a bifurcation between more economically liberal societies, which tend to pursue dualist rental strategies, and more or less social democratic societies that pursue unitary ones. Research inspired by this approach often tends to emphasize the negative outcomes of market forces in housing, such as social segregation and marginalization, along with the positive aspects of unitary systems as forces for social inclusion and equality. Our data and analyses below also indicate increasing economic polarization and spatial segregation resulting from the transition from the former system to the latter in Amsterdam. The concern, nonetheless is not a normative one, but rather, to illustrate the salience of urban transformations and the development of this theoretical model in contemporary context.

### Unitary and dualist rental markets

In countries featuring a dualist housing approach, the state typically maintains a controlled social rental sector in which strict means-tested access for households is applied. This regulated sector is kept small to discourage its use and promote the private market for housing. Through state intervention, social rental housing thus becomes a ‘safety net’ or even an ‘ambulance service’ (Stephens and Fitzpatrick 2008), often reserved for the poorest or most marginalized households. Within these systems, the private rental sector tends to be sheltered from non-profit forms of housing provision and functions along market lines. The result, as Kemeny (1995) puts it, is ‘that a curious dualism emerges between a largely unregulated profit-driven [private] market (…) and a tightly controlled state sector’ (p. 9). Renting overall often becomes unattractive, with the non-profit rental sector particularly stigmatized due to its marginalization and means-tested access, and the private rental sector because of high rents and limited tenant protection. In these ‘split’ rental markets, therefore, owner-occupancy normally constitutes the mainstream tenure.

In the unitary model, on the other hand, the state is inclined to structure the market in order to encourage competition between non/limited-profit (social) and for-profit (private) rental housing. This competition can be considered successful in unitary systems when households are able to choose freely between non-profit and for-profit rental housing without having to compromise on price or quality. Non-profit housing landlords typically provide cheaper housing, and thereby influence market rent levels (effectively forcing for-profit landlords to lower their rents). Importantly, this dampening effect on rent levels is more pronounced when the non-profit sector has a substantial market share, is accessible to a broad range of income groups and when the quality of its stock is high. Housing policy in unitary rental markets is typically tenure neutral. Nonetheless, as both types of rental housing are attractive and accessible to households across income classes, demand for owner-occupied housing is weaker.

Kemeny et al. (2005) later advanced the unitary/dualist typology by distinguishing between *unitary* and *integrated* rental markets. Integrated rental markets can be considered a late or final stage of a unitary rental market, as over time non-profit housing providers undergo a process of maturation. In the initial stages of development, while there are no regulatory barriers to competition between non-profit and for-profit renting, the non-profit sector is still largely uncompetitive because of its debt burden. State support via subsidies and tax advantages initially sustain immature non-profit sectors ensuring its survival, but later ‘develop it to the point at which it can begin to offer real competition across the whole rental market’ (Kemeny et al. 2005, p .859). The process of maturation is principally based on the solidity (equity–debt ratio) of non-profit organizations, which is believed to increase over time through amortization of debt and increases in market values (Kemeny 1995; Elsinga et al. 2008). Non-profit housing providers, as they mature and become more competitive ‘can translate their willingness to accept a lower return on their relatively large equity into lower rents (…) that shadow those of a profit rental market….’ (Elsinga et al. 2008: p. 24). Maturation can also be understood as part of the evolution of unitary rental markets. In the later stages, non-profit providers achieve financial independence and can vie with for-profit renting on relatively equal terms, with the rental sector as a whole thus becoming ‘integrated’.

By investigating developments in unitary rental markets in the Netherlands, Sweden and Switzerland, Kemeny et al. (2005) found, despite clear signs of maturation, each differed in the degree to which they were moving towards integration. More importantly, Kemeny et al. tentatively described retrograde features (i.e. *dis*integration) that could potentially undermine the process of maturation. While the authors downplayed counter-developments, if we consider more recent advances in some of these housing systems, ‘retrogression’ takes on a more significant hue. Specifically, since the 2000s, there has been growing evidence of transition from unitary to more dualist arrangements, especially in terms of the increasing assertion of market practices and preferences for home ownership.

### Retrogression and threats to the unitary rental market

In recent years, governments in most European countries have more actively implemented market-oriented housing policies and sought to transform the tenure structure of housing markets, not only stimulating home ownership, but also forging a split between social and private rental housing in many cases (see Christophers 2013; Kadi and Ronald 2014). Indeed, following a neoliberal rhetoric, home ownership has been increasingly portrayed as ‘natural tenure’ across Europe that offers more autonomy to the occupier as well as greater economic security (see Doling and Ford 2007). Subsequently, state-regulated or state-owned social rented housing has sharply declined while owner-occupation rates increased across most continental European countries (Czischke 2009, Fitzpatrick and Pawson; 2014; Priemus and Dieleman 2002; Larsen and Hansen 2015).

Elsinga et al. (2008) have further argued that ‘the growth of home ownership is gradually eroding the unitary model’ (p. 35), suggesting that as government policies have shifted their focus to owner occupation and housing privatization, unitary rental systems, even in social democratic regimes, have lost tenure neutrality. In some contexts this has been driven by policies supporting sales of non-profit rental dwellings and greater concentration of low-income groups in an increasingly shrinking social rental sector (see Forrest and Murie 1988; Malpass 2005; Van Kempen and Priemus 2002). In these contexts, the roles of different tenure sectors have shifted, with the private rental sector designated as an intermediate sector for households who will or cannot yet buy, and a social rental sector targeted, increasingly, at low-income and vulnerable households.

### Dualisation and social change

Although processes of unitary system retrogression are often alluded to, they have been under researched with greater emphasis often placed on maturation and path dependency (Norris 2014). We argue in this paper that dualisation itself is a critical process with key outcomes that inform wider debates on spatial and socioeconomic polarization. Whereas unitary models can reduce social segregation and prevent the stigmatization of social housing, dualist models are argued to deepen social inequalities and help institutionalize a disadvantaged underclass (Kemeny 1995; Fitzpatrick and Pawson 2014). Little attention, however, has been paid to the transition from the former to the latter, or the particular outcomes.

A specific consequence of the split forged between social and private housing under dualisation, is that the social sector is typically reduced to the status of ‘safety net’, and increasingly reserved for the weakest and most marginalized (Forrest and Murie 1988). Subsequently, when neighbourhoods associated with social housing become more marked, this stigma becomes territorial. The social consequences of living in stigmatized neighbourhoods of concentrated poverty are well-researched (e.g., Wacquant 2008; Galster 2007; Pinkster 2009), and a recurring theme in urban policy throughout Europe and North America (e.g. Andersson and Musterd 2005; Goetz 2003). Yet, despite knowledge of, and programs to deconcentrate poverty, interventions largely fail to either consider or address the unraveling of unitary rental markets.

Increases in social inequality have also been associated with housing market restructuring in, for instance, Sweden (see Christopers 2013) and the UK (see e.g., Hamnett 2003). In these contexts privatization and neo-liberalisation have dominated debates rather than dualisation as a process. In Sweden, large-scale sell-offs of public housing stock, the subsequent expansion of co-operative housing (*bostadsrätt*) and the move away from tenure neutrality had very distinct societal outcomes. In Sweden, tenure restructuring helped aggravate segregation and socio-economic polarization, and contributed significantly to the marginalization of public housing (Andersson and Turner 2014), especially in central urban areas. In the UK, market-oriented housing policies were implemented earlier and more fiercely (Forrest and Murie 1988), shaping urban socioeconomic inequalities and undermining housing conditions of lower-income households in particular (Hamnett 2003). Dualisation, and not simple privatization, however, has also been central to transformations in both contexts and, as we will now argue using the example of Amsterdam, connects policy reforms, as well as tenure restructuring, more directly with social and spatial outcomes.

## The Dutch unitary rental market model

### Characterization of the Dutch housing system

In their 2008 analysis of the Dutch rental system, Elsinga et al. (2008) concluded that the Netherlands, in line with Kemeny’s (1995) original classification, could still be considered a unitary rental system, although this status was under threat. The unitary nature of the Dutch housing system is mainly observed in its large and broadly-accessible social housing sector. In 1985, almost 40% of all dwellings in the Netherlands belonged to the social housing stock (Van der Cammen and De Klerk 2003). While this had fallen back to around 32% by the mid-2000s, Dutch social housing still represented the largest and most distinctive non-profit housing sector in Europe (Whitehead and Scanlon 2007). As its meaning can differ across different context, the term ‘social housing’ requires some unpacking in the Dutch case. Conventionally, social housing has been defined as ‘residential accommodation provided at submarket prices by the state or non-profit landlords and allocated according to administrative criteria rather than price’ (Fitzpatrick and Pawson 2014, 598). In the Netherlands, the state itself is not a direct provider of housing (social housing is thus not public housing), with this task carried out primarily, but not exclusively by independent, not-for-profit housing associations. Critically, although most social rental housing is owned by non-profit housing providers, certain cities have a substantial share of privately owned, rent regulated (but for-profit) social housing. In Amsterdam, the social housing sector conventionally consists of all rental units with rents under € 710,- which is the so called *liberalisatiegrens (liberalization line).* Dwellings with higher rents constitute the free market rental sector (subject to controls on rent increases) and can also be provided by for-profit and, more recently, not-for profit providers. In Amsterdam, almost 50 thousand private rental units are rent controlled (around 12% of all housing). These units are treated the same by local authorities as the non-profit lettings of housing associations.

Social renting in the Netherlands has long been an attractive alternative to private housing (owner occupancy or private rent) and has included large middle-income segments of the population. Its success largely derives from broad access, high quality housing stock and security of tenure (Meusen and Van Kempen 1994; Murie and Musterd 1996). In recent years, the scale of social rental housing provision has diminished, but still represents around 30% of the national housing stock (Boelhouwer and Priemus 2014). Moreover, in the larger cities like Amsterdam and Rotterdam, social rental housing still represents the largest sector by far (at 44% in Amsterdam and over 50% in Rotterdam in 2015).

Historically, the national government has orchestrated the housing sector to ensure affordable and accessible housing for all households. Through the support of secure, low-cost housing, households have been expected to maintain a standard of living independent of their income. Social rental housing provision has not only contributed to this directly, but also indirectly by dampening private demand and thus private rent prices (Musterd 2014). In either case, both left and right wing parties, usually as coalitions, supported the construction of social rental housing for much of the twentieth century, which not only maintained social welfare but also sustained urban growth and renewal. This was primarily achieved through support of quasi-independent housing associations, which became particularly active in the post-war decades (Van der Schaar 1987). Nonetheless, since the 1980s, support for more market-based, commodified forms of housing provision (especially home ownership) has grown, and since the 1990s has advanced further along with the transformation of housing associations into private, not-for-profit organizations (see Van Kempen and Priemus 2002).

### Undermining the unitary rental system

The last decade however, has witnessed some remarkable transformations in Dutch housing, with government policies adapting reactively to housing market and economic volatility, challenging the sustainability of the unitary rental market. A number of specific and interlinked attacks on the Dutch unitary model can be identified, many of which originate in how social housing is regulated and controlled, as well as how social housing is being renegotiated and politically charged.

Since the quasi privatization of Dutch social housing providers in 1995 (*ibid*.), housing associations have been independent, non-profit institutions subject to a legal requirement to support affordable rental housing for market excluded households. Associations had their public debts written off and became the direct owners of their real estate assets. Under the new financing model, housing associations also became dependent on sales and other commercial activities. Specifically, in order to finance the construction of new social housing and support urban renewal projects, associations were to make use of income generated from project development as well as from sales of existing social rental units (see also Aedes 2013). In terms of their operations, social housing providers thus became hybridized: somewhere between state and commercial organizations with competing social and market logics (see Blessing 2012).

However, this hybrid status later gave rise to complaints from commercial organisations to the European Commission concerning unfair competition between housing associations and private developers (see Priemus and Gruis 2011). The Dutch government responded by imposing new rules on housing associations in the late-2000s, which became even more restrictive with the election of a more economically liberal political coalition in 2010 (the first Rutte cabinet). Critically, concessions for non-profit housing associations were to be limited to their social activities, while their commercial undertakings were to be treated as market competitive and subject to corporate tax. This required housing associations to split their stock into more defined commercial and social parts, with social landlords subsequently circulating part of their stock in the market-rent sector (see Hochstenbach 2017). New legislation also stipulated that 90% of new social rental allocations should be to households with an income below a specified limit.^1^ This introduced a more restrictive, means-tested type of housing allocation and made the sector far less competitive with private sector housing. The stricter entitlement system has also contributed to the social sector homogeneity in terms of tenant composition (see below).

Another critical change in the status of housing associations was the Housing Agreement (*Woonakkoord*) of 2014. Since the 2000s, Dutch governments have increasingly shown interest in deploying the considerable financial reserves of housing associations. The most recent manifestation thereof is the ‘landlord levy’ imposed on landlords of properties with regulated rents (i.e. housing associations), which is expected to generate extra annual tax revenue of around € 1.7 billion. The same legislation also allowed social landlords to raise rents (up to 6.5%) above inflation rates for households with incomes above the new low-income limit in order to help meet the new charge. This has arguably undermined the rent damping effect of social on private rent levels. Moreover, Priemus (2014) has argued that the levy will have far-reaching consequences for housing associations as independent, non-profit organizations, with the withdrawal of capital diminishing their position in the market.

### Privatization of social housing and focus on owner-occupancy

Another key threat to the Dutch unitary rental system has been the privatization of large numbers of social housing units. The promotion of home ownership since the 1990s has both reflected and reinforced diminishing support in Dutch politics for the unitary rental model (Aalbers 2004; Boelhouwer and Priemus 2014). The 2000 landmark policy statement (*nota*), *Mensen, Wensen, Wonen*, set out a ‘mission’ to achieve a national home ownership rate of 65% by 2010. To attain this goal, about 700,000 rental units^2^ would need to be converted: i.e. 162,000 private rentals units and 538,000 social rental ones (a reduction of about 21% and 25%, respectively). These aims were framed around discourses that asserted that home ownership would enhance individual autonomy and responsible social participation as well as potential asset accumulation (Van Gent 2013).

Policies in the early 2000s strongly supported this aspiration with housing associations encouraged to sell-off a significant portion of their stock. Initially, housing associations were slow to change and by 2004 less than half of expected tenure transfers had been realized (see Aalbers 2004). Sales of newly built units provided by housing associations were often more effective in increasing the owner-occupied stock. Nonetheless, by the mid-2000s, as support for the unitary rental system began to fall away, the conversion of social rental housing to owner-occupation began to intensify. Indeed, in the last few years the government has become increasingly vociferous in demanding that housing associations sell off their dwellings (Parool 2016).

While tenure neutral housing policy is considered central to the success of unitary rental systems, recent pro-home ownership policies have helped undermine the balance between private and social sectors. Indeed, the relationship between buying and renting has become particularly ‘lopsided’ (see Priemus 2014). During post-crisis rounds of government spending cuts, support for home ownership has, notwithstanding some adjustment to tax deductions for home buyers,^3^ fared rather well. Meanwhile, individual rent subsidies have been reduced along with the stricter targeting of very low-income households. In the Housing Agreement of 2014, the government also expressed an intention to ‘persuade’ better-off households to move out of the low-cost, social rental housing.^4^

With the stock residualised and better-off tenants being pushed out, social housing in the Netherlands is becoming the de facto tenure for the poor and marginalized. Critically, with increasing concentrations of low-paid and unemployed tenants along with declining market share, it is losing its competitive hold. Owner-occupation meanwhile is ostensibly being reconstituted, normatively and materially, as the mainstream tenure.

## The Dualisation of Amsterdam

Considering the restructuring of social housing in the Netherlands, there appears to be considerable support for the notion that the unitary rental system is unravelling. In, Elsinga et al. 2008 envisaged two possible scenarios for the Dutch housing system. In the first, the unitary rental system survives on the basis that housing associations continue to be well-funded (matured) and the portion of social rental housing remains above 30%. The second anticipated a ‘residual unitary market scenario’ (see Van der Heijden 2002) in which housing associations became ‘hybrid’ organizations and social housing acquired a safety-net function. The latter scenario, while roughly sketched, currently seems the more salient.

While Elsinga et al. considered the housing system in terms of national developments, our concern is with a more specific context and how transformations in the unitary rental system are actually manifesting. The municipal district of Amsterdam provides a particularly relevant case. On the one hand, it represents a ‘just city’ (Fainstein 2010), supported by the strong unitary character of Amsterdam’s housing market. On the other, Amsterdam is strongly interconnected to the global economy and a particularly dynamic market context (Engelen and Musterd 2010).

Our analysis begins by establishing how housing reforms have impacted Amsterdam specifically—where housing associations are the main providers of housing—highlighting divergence with the national picture in terms of unitary model unravelling. We further address key developments in the spatial distribution of tenure between neighbourhoods, as well as the more marked redistribution of the social housing stock between the centre and periphery, as distinct outcomes of dualisation. Our final analysis considers dualisation in terms of (1) rent levels and (2) tenant (income) profiles, drawing on household survey data^5^ (1995–2013). While we demonstrate housing system polarization—with private housing (especially home ownership) becoming increasingly mainstream and social rental housing more concentrated in terms of low-income households (i.e. residualizing)—we more critically show how private and social tenant profiles have diverged in the last two decades, which we argue is indicative of the waning state of the unitary rental system.

### Recent developments in the ‘just city’

In the Netherlands the social housing sector accounted for almost 40% of all housing by 1989, after which the sector began a gradual decline to approximately 32% by 2014. Amsterdam has followed a different pattern with social housing peaking at 58% of housing in 1995 before falling to 44% by 2015 (O&S 2015). Moreover, as approximately half of private rental stock is also rent regulated, taken as a whole, the ‘socially regulated rental housing sector’ accounted for more than two thirds of Amsterdam homes in the mid-1990s.^6^ Meanwhile, the share of owner-occupied housing stood at just 11% (*ibid*). By international standards, the social rental sector also offers high-quality, low-rent housing accessible to diverse income groups (Musterd 2014; Boterman and Van Gent 2014). Due to its scale, the socially regulated rental stock has overshadowed the free market rental sector, with prices in the former strongly influencing the latter. As such, Amsterdam at the turn of the century represented a mature and relatively robust unitary housing system. Furthermore, social rental housing was spread relatively evenly across the city—even in high status neighbourhoods like the central canal district—restricting segregation (Fainstein 2010).

The capital’s highly-regulated and de-commodified housing system demonstrated a strong focus on diversity, equality and accessibility for all (Fainstein 2010). However, the threats to the unitary rental system, already evident at the national scale, began to gain a foothold in Amsterdam around the turn of the century (Aalbers 2004). The national push for home ownership and diminishing state support for social rental providers arguably had the most direct impact on Amsterdam’s local market. In line with national policy, the Municipality of Amsterdam set out to explicitly increase the share of owner-occupied housing and slowly started to privatize, liberalize and deregulate parts of the city’s housing stock (see Uitermark 2009; Van Gent 2013).

**Fig. 1:**
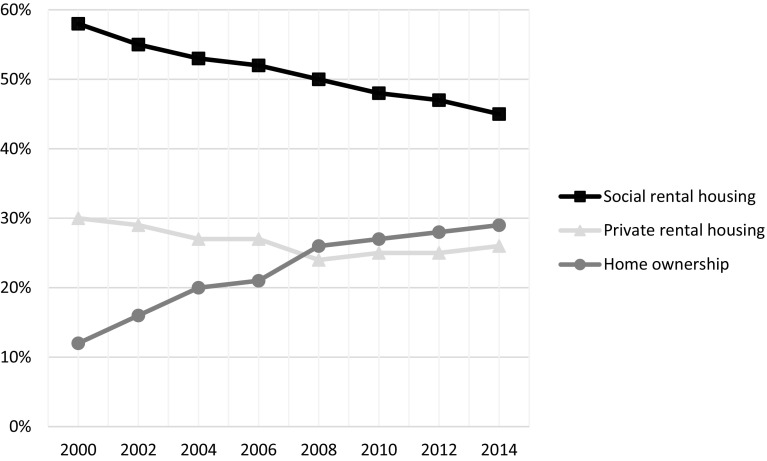
Tenure developments in Amsterdam between 2000 and 2014. Tenures displayed as a percentage of the total housing stock. *Source*: Amsterdam Bureau of Statistics 2015

Figure 1 provides an overview of Amsterdam’s housing tenure structure and its development since 2000. Home ownership rates have nearly doubled with owner-occupied housing increasing by more than 52.000 units. Both sell-offs of social housing and new-build have contributed to this rapid increase in owner occupied units, although social rental housing remains, for now, the dominant tenure in the city. Private renting, meanwhile, lost around 5% sector share between 2000 and 2008, but subsequently reversed its decline. Since the start of the GFC, the share of the private rental sector has gone up by nearly 3%. We will come back to the details of this marginal, but meaningful trend, as it plays a substantial role in dualisation processes.

In addition to national policy discourses espousing the social, economic and ontological benefits of owner occupation, tenure conversions in Amsterdam were also central to municipal regeneration strategies that sought to modernize and diversify city neighbourhoods. Indeed, during this period the city ostensibly adopted an aggressive gentrification strategy with the objective of attracting higher-income households to lower-income neighbourhoods (Van Gent 2013). The policy discourse asserted that tenure conversions, from rent to owner-occupation, would attract more affluent groups seeking opportunities to buy, with beneficial social effects for poorer districts in terms of social mix (Boterman and Van Gent 2014).

As owners of around half of urban housing (and the majority of the rental stock) since the 1980s, housing associations have been key actors in the privatization process. Unlike tenure conversions in other countries, however, there has been no ‘Right to Buy’ policy (as in the UK) for sitting tenants in the Netherlands. There is instead a ‘Right to sell’ (see Aalbers 2004) with individual housing associations deciding whether sitting tenants can buy, or if a vacant property should be sold. While there have been no legal measures forcing housing associations to sell, there has been growing political pressure on them to do so (Uitermark and Bosker 2014). Sales were also necessary under the revolving fund system in order to acquire the necessary finances to construct new dwellings (Gruis et al. 2004). More recently however, the state has more effectively compelled municipalities and housing associations to unload social rental housing units on the market, especially since the GFC and the introduction of EU-proof rules on social housing allocations. Locally, a series of agreements,^7^ were negotiated by the municipality, district authorities and housing associations between 1998 and 2007, allowing for a total of 28,575 social rental units to be sold to home buyers. In the extension of the agreement (2008–2016), the privatization quota was increased to over 30,000 units: approximately 15% of the city’s social housing stock (Municipality of Amsterdam 2008).

### The geography of dualisation

As the analysis of Amsterdam above illustrates, dualisation is a more nuanced and uneven process than traditional housing studies have accounted for, with the roles of, and relationships between, different actors and institutions framing transition. Moreover, as we now consider, there are also significant geographical factors that shape housing market dualisation that contribute to inequalities across the city and between neighbourhoods.

**Fig. 2:**
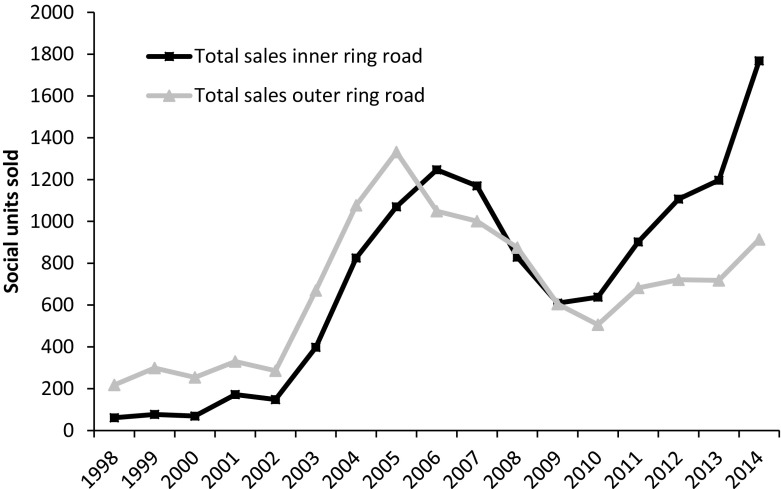
Sales of social rented dwellings by housing associations, 1998–2014. *Source:* Amsterdam Federation of Housing Associations AFWC (2014)

Figure 2 illustrates the scale of sales of social rented housing in the period 1998–2014, during which 23,824 social housing units were sold-off (AFWC 2015). These sales however, have not occurred evenly over time or across space, with dualisation shaped by particular spatial constraints. For Amsterdam’s housing market, the ring-road plays a key role in this process, representing a physical boundary between pre-war and post-war built environments, as well as between high- and low-density housing. From 1998 up until 2005, social housing sales outside of the ring-road in the northern, south-eastern and western periphery exceed sales inside the ring-road and central areas of the city. Amsterdam’s social housing sector is most dominant beyond the ring-road, and sales in these areas have contributed significantly to greater diversification of the housing stock there. Housing associations were initially reluctant to sell inner city units since this property was more prized by associations and, moreover, sales would not contribute to greater diversification of the stock.

Between 2010 and 2014, however, the approach of housing associations appears to have changed with social housing sold off in inner city districts intensifying (see Hochstenbach 2017). Indeed, by 2014 housing associations were selling nearly twice as many inner city units.

**Fig. 3:**
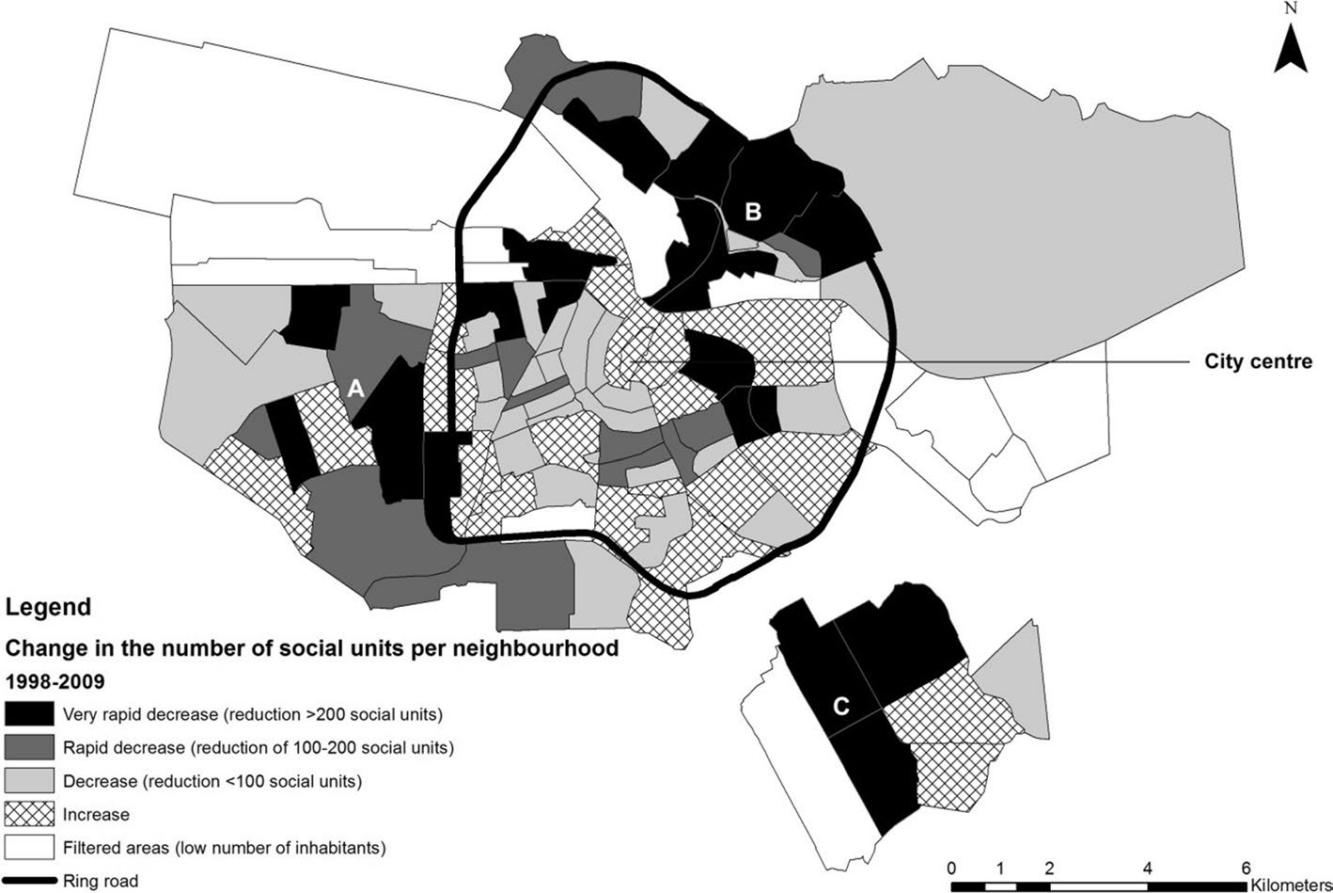
Transformations in the distribution of social rental units per neighbourhood in Amsterdam, 1998–2009. District (A) = Nieuw-West, (B) = Noord, (C) = Zuidoost. *Source*: Amsterdam Bureau of Statistics, 1998–2009.

**Fig. 4:**
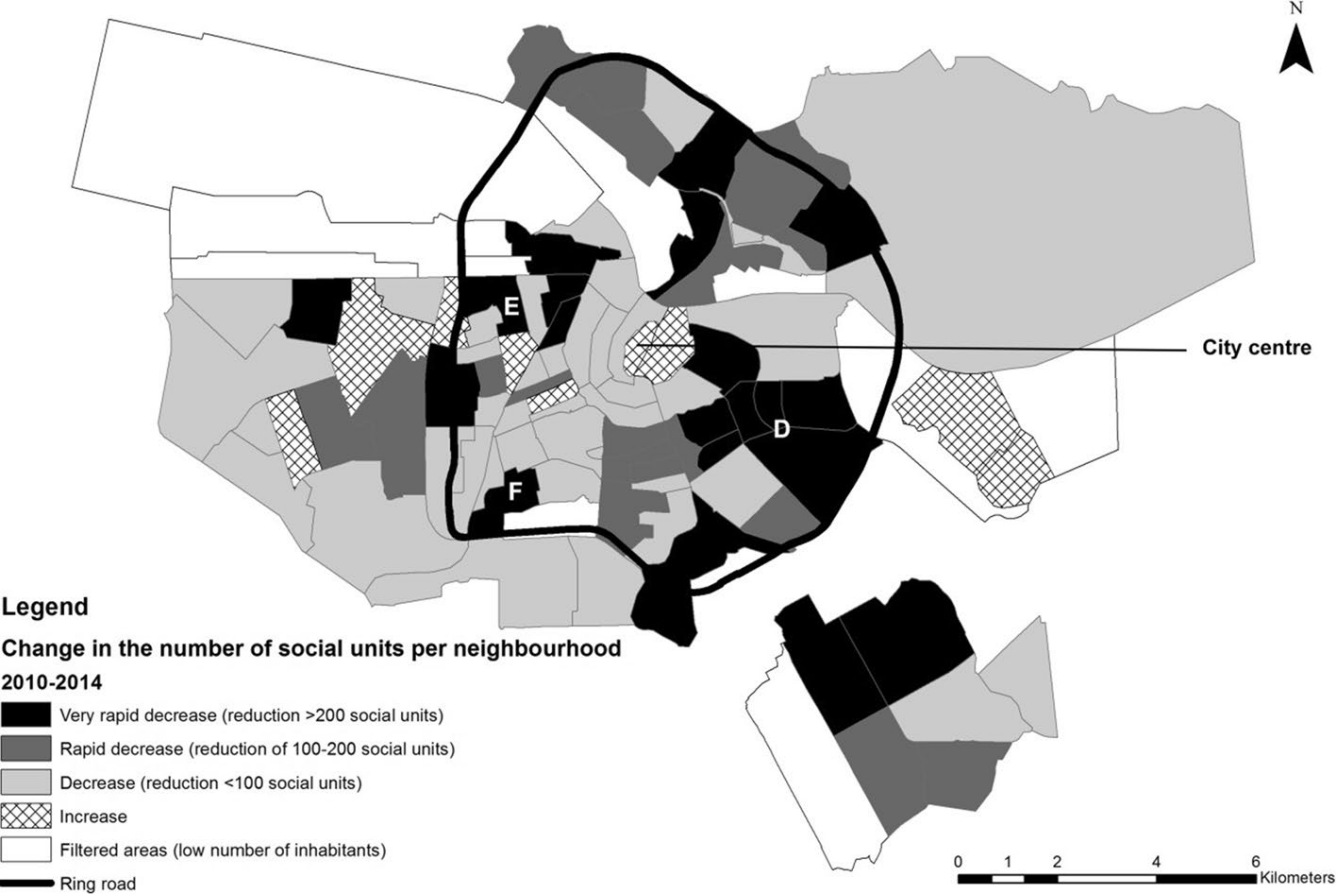
Transformations in the distribution of social rental units per neighbourhood in Amsterdam, 2010–2014. District (D) = Oost, (E) = West, (F) = Zuid. *Source:* Amsterdam Bureau of Statistics, 2010–2014

A more detailed geospatial analysis confirms the shift in the geography of social housing privatization in recent years. Figure 3 specifically illustrates relative increases and decreases in the distribution of social rental units per neighbourhood in the period 1998 to 2009. The map demonstrates marked decreases in the outer peripheral areas during this period, especially the sub-districts of Nieuw-West (A), Noord (B), and Zuidoost (C). Strikingly, most inner-ring neighbourhoods show (slight) increases in numbers of social dwellings, i.e. additions to the social stock exceeded sell-offs in these areas between 1998 and 2009. Figure 4 illustrates transformations in the distribution of social rental units per neighbourhood in the period 2010 to 2014. Neighbourhoods that show increases in the number of social rental units in the inner-ring have almost disappeared. Instead, large clusters of ‘very rapid decreases’ and ‘rapid decreases’ have formed, especially in the more central sub-districts of West (E), Oost (D) and Zuid (F). Peripheral districts, on the other hand, have slower rates of decline in the more recent period. This is arguably due to the growing imperative to comply with the landlord levy—with associations capitalizing on the ‘value gap’, which is the biggest in the inner city—as well as the emerging role of housing associations in privatizing ‘excess’ social stock in high-demand locations (high-value) and not just low-income rental neighbourhoods.

Thus, over time the focus of conversions has shifted from the outskirts to the middle, with housing market restructuring stimulating urban polarization, especially between inner- and outer-city neighbourhoods. Under the latest round of policy reforms, housing associations are more likely to sell-off their best homes. The social sector is thus not only losing market share, it is also being stripped of its best quality housing, especially in the more attractive areas of the city, with stock becoming more concentrated in post-war apartment blocks on the periphery. Emerging patterns of tenure appear to reflect an intensive shift from a unitary toward a more dualist rental system. This shift is also contributing, moreover, to greater owner-occupation and deeper housing commodification in the inner city, especially since 2009/2010. Essentially, the capacity of the social sector to provide good quality housing and effectively influence prices in the private sector, key to unitary systems, is being eroded.

### Developments in rents and tenant profiles

As we established earlier, an important criterion of the unitary model is the extent to which both social and private rental sectors function either as equal, integrated sectors, or demonstrate a significant cleavage (as in dualist systems). In Amsterdam, we can further explore signs of dualisation in terms of both rents and tenant income profiles. Critically, we now consider whether the dampening effect of the social sector on market rent levels is diminishing as well as divergence in social and private tenant profiles.

**Fig. 5:**
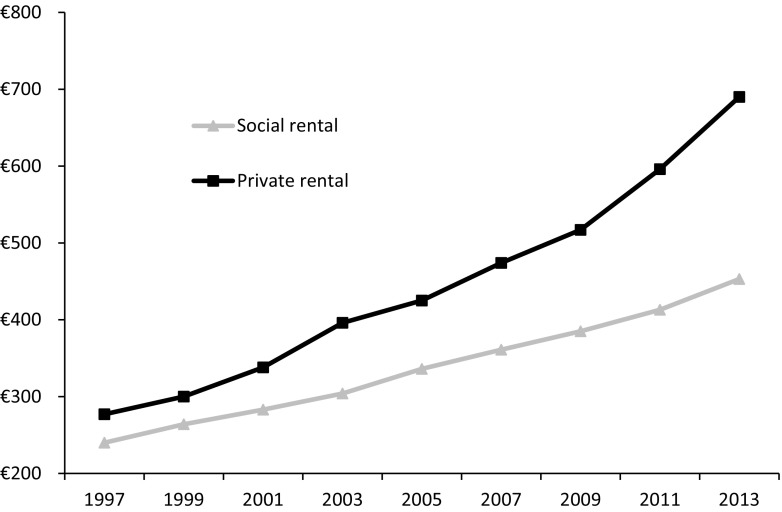
Average rental price in the social rental sector and private rental sector in Amsterdam. *Source*: Living in Amsterdam survey, 1995–2013

According to City of Amsterdam data, average private sector rents in 1995 were comparable to social sector ones: € 277 versus € 240, respectively (see Fig. 5). The large and broadly accessible social rental sector seems to have had a dampening effect on market rent levels, and rents were highly comparable. This also suggests a high degree of competitiveness in terms of rents and quality, characteristic of a unitary rental market. However, since then, rents in the private rental sector have rapidly increased, especially after 2009. The average private sector rent is now almost double that of the social sector, representing a marked discrepancy in affordability.

There are, arguably, two explanations for the increasing divergence in rents between sectors. First, after the GFC, mortgage lending criteria tightened, decreasing household borrowing capacity (see Ronald and Dol 2011). Many households could no longer access home ownership and turned to the private rental market instead, increasing demand (Municipality of Amsterdam 2015). Insecurity in both housing and employment markets may have also reinforced the attractiveness of renting. Another factor that has to be taken into consideration has been the increasing flow of investment in the private sector in Amsterdam since the GFC. Indeed, recent data indicate that Amsterdam appears to have become the target of buy-to-let investment practices (ING 2017). Linking back to Fig. 1, these practices have increased the share of non-rent controlled private sector lettings. With home ownership becoming too expansive, new arrivals in Amsterdam, with higher incomes are increasingly being ‘pushed’ into this sector. Second, while research shows that social rental housing is highly affordable, it is also much less accessible, featuring increasingly longer waiting lists^8^ (Uitermark 2009; Kadi and Musterd 2015). In effect, declining access is undermining the dampening effect of the social sector on private rents, exacerbating disparity between sectors.

With the decrease in social housing, the recent targeting of lower-income households and households on benefits, and increasing disparities between rents in private and social sectors, the profile of dwellers in each housing sector is rapidly diverging. This becomes most evident if we look at real income developments among households across rental sectors. Table 1 shows that in 1995 the median real monthly income of a private rental tenant in Amsterdam was only slightly higher than that of a person in social rental housing. Both rental sectors appear to have had considerable parity and together provided affordable housing for similar low and middle-income households, a key feature of a unitary rental market. In less than two decades, however, much has changed, as the inflation-adjusted income of private renters has increased by 53%, compared to only 9.6% for social renters. Indeed, while private renting has become the domain of middle-income people, social rental housing is increasingly the sector where lower-income households end up. Owner-occupation continues to serve largely higher-income groups.

**Table 1:** Developments in median real monthly income of Amsterdam renters and home owners, 1995–2013, (inflation-adjusted) *Source*: living in Amsterdam survey, 1995–2013; CBS Statline, 1995–2013.

Year	Social rental housing	Private rental housing	Owner-occupied housing
1995	€ 1531	€ 1684	€ 2862
1999	€ 1578	€ 1901	€ 2971
2005	€ 1695	€ 2292	€ 3685
2007	€ 1755	€ 2411	€ 3653
2009	€ 1776	€ 2468	€ 3694
2011	€ 1667	€ 2500	€ 3655
2013	€ 1678	€ 2573	€ 3646
Total increase 1995-2013	€ 147	€ 889	€ 784
Total increase in %	9.6%	53%	27%

## Discussion and concluding remarks

The Netherlands has historically been understood to have had a strong unitary housing system, contributing significantly to socioeconomic equality as well as ‘justice’ for different varieties of urban citizen (i.e. Fainstein 2010). However, as we have illustrated, the Netherlands seems to have drifted toward a dualist housing system in which social and private sectors are critically unequal; undermining the capacity of non-market housing providers to keep rents and housing costs low for the populations as a whole. We considered both the momentum and direction of social and private housing market transformations in recent years, identifying an intensification in both the rate of change and the most recent policy interventions. While various privatization and marketization strategies have driven housing sector restructuring, it is the unraveling of the unitary framework, we argue, that has shaped particular social and urban changes, and sustained the ascension of dualism.

Our analysis has moved on from previous, more generalized observations made in the pre-crisis era (e.g. Van Kempen and Priemus 2002; Elsinga et al. 2008), to provide a more definitive picture of ongoing transition from unitary to dualist practices in the Netherlands. By taking Amsterdam as an empirical focus, this paper has also gone beyond traditional housing studies analyses to reveal how dualisation unfolds and has particular spatial outcomes. On the one hand, we show that the promotion of home ownership and the re-alignment of housing associations has stimulated greater polarization of social and private housing, especially between inner and outer ring-road neighbourhoods. If this trend continues, housing associations are likely to be left with mostly the least desirable housing stock (especially post-war concrete flats), concentrated largely on the outer urban periphery. On the other, our analysis of household income, rent and tenure shows that both private and social sectors in Amsterdam are playing increasingly different and unequal roles. Social rental housing is being residualized in terms of size and the profile of its tenants. Moreover, the socio-economic gap between tenants in the social and private rental sectors has increased. The social housing sector is still large and typically affordable, but increasingly more difficult to access. Meanwhile the private rental sector along with owner-occupied housing, has become more expensive and increasingly comprised of better-off households.

There are various implications of this analysis for both studies of housing systems and cities, and for understanding recent social changes. Firstly, looking back on speculations from almost a decade ago, it seems far clearer that the Netherlands is on a route toward a more neoliberal housing model. Indeed, it is quite possible that Dutch home ownership rates may soon overtake those of English speaking nations associated with dualist markets, such as the UK and USA. While Elsinga et al. (2008) deliberated on two potential scenarios, the unraveling of the unitary model is now much more obvious, with differences between social and private sectors in terms of prices and occupants, as well as associated locations, becoming more marked. Moreover, the *retrograde* developments in unitary systems downplayed by Kemeny et al. (2005) now appear much more significant. While the ‘maturation’ of the social housing stock had been taken to imply considerable sector robustness, the path dependence of ‘integrated’ housing systems (Kemeny 1995) seems to be somewhat more fragile than envisaged. Nonetheless, at the time of writing of this article, new local government guidelines have been mooted to dampen escalating housing in-affordability in Amsterdam, with new output focused on both low and middle income renters (NRC 2017), suggesting history is important to housing systems and that neo-liberalization is a particularly uneven process (see also Norris 2014). Indeed, since 2018, caps have been put on social housing sales (at a maximum of 1200 a year), and a line has been drawn under the minimum amount of social units in Amsterdam (approximately 162,000 units).

Secondly, we argue that dualisation is a critical process, especially in the ways in which cities are being socially made and re-made. While debates over the ‘just city’ and urban inequalities have been dominated by gentrification perspectives (Préteceille 2007; Lees 2014), displacement (Atkinson 2000; Slater 2009) and neoliberalisation (Musterd 2014; Kadi and Musterd 2015; Van Gent 2013), dualisation is vastly overlooked, but, as we argue here, is an important lens for understanding contemporary urban and social change. In this observation also lies an important theoretical contribution of the paper since the transitioning of unitary to dualist systems, and the particular social outcomes thereof, are still vastly under researched. We showed that the unbalancing of private and social housing sectors (i.e. dualisation) has had very distinct spatial outcomes, deepened class differences and become a threat to Amsterdam’s status as a just city. Further research with more nuanced socioeconomic variables (e.g., level of education, employment, etc.) is ultimately necessary to validate our findings concerning socio-economic changes at the micro-household level. Nonetheless, our results and specific rental system approach are, arguably, significant for other urban contexts, both in the Netherlands and abroad. Tentative evidence across Europe suggests that most housing systems have become characteristically market-orientated (see Federcasa 2006; Ghekière 2007; Van Kempen and Murie 2009), and are thus geared toward dualisation. For scholars concerned with social justice (e.g., Purcell 2002; Gilderbloom and Hanka 2007; Uitermark 2009; Fainstein 2010; Uitermark and Bosker 2014), the unbalancing of social and private sectors can help to foreground and inform these debates.

## Footnotes

1 This limit was initially set at € 34,085, but subsequently increased to € 34,911. Personal income (median gross income) in the Netherlands was € 36,500 in 2016 (CPB 2016).

2 Equivalent to around 10% of the total housing stock of the Netherlands at the time.

3 It is estimated that Mortgage Interest Tax Relief (*hypotheekrenteaftrek*) costs Dutch government more than € 14 billion per year year (Conijn 2008). Since 2011 however, steps have been taken to gradually reduce tax advantages, especially for new home-buyers.

4 Including the requirement to raise rents in the regulated (social) stock occupied by better-off households (income above € 43,000) by up to 6.5% p.a.

5 The Living in Amsterdam (WiA) survey (N = 18,920) is a major biennial survey of housing sectors and conditions. It also provides data on income group distribution across these segments.

6 Depending on dwelling size and quality, rental housing is classified as (1) regulated or (2) unregulated, regardless of the status of the owner. Regulated dwellings have rents < € 710, and are mostly, but not exclusively, socially owned (housing associations). However, in Amsterdam there is still a significant share of privately owned, rent regulated housing. Unregulated rental dwellings (‘free market rental sector’) have rents > € 710. These are mostly privately owned, although housing associations now also operate in this segment.

7 Social Housing Sales Covenants I and II.

8 The average waiting time for a two-bedroom apartment in Amsterdam’s social rental sector in 2016 was around 9 years, up from 5.5 in 2007 (AFWC 2014).
